# Supplementary material for: Interactions of Nucleosomes with Acidic Patch-Binding Peptides: A Combined Structural Bioinformatics, Molecular Modeling, Fluorescence Polarization, and Single-Molecule FRET Study
Source: Int J Mol Sci. 2023 Oct 14;24(20):15194. doi: 10.3390/ijms242015194 (PMC10606924; doi:10.3390/ijms242015194)
Supplement: Supplementary file 1 [file ijms-24-15194-s001.zip › ijms-2655940-supplementary.pdf]

Supplementary Information

Interactions of Nucleosomes with Acidic Patch-Binding Peptides: A Combined Structural Bioinformatics, Molecular Modeling, Fluorescence Polarization, and Single-Molecule FRET Study

Pavel D. Oleinikov[1,†], Anastasiia S. Fedulova[1,†], Grigoriy A. Armeev[1,†], Nikita A. Motorin[1,†], Lovepreet Singh-Palchevskaia[1,†], Anastasiia L. Sivkina[1,2], Pavel G. Feskin[1], Grigory S. Glukhov[1,3], Dmitry A. Afonin[1], Galina A. Komarova[4], Mikhail P. Kirpichnikov[1,5], Vasily M. Studitsky[1,6], Alexei V. Feofanov[1,5] and Alexey K. Shaytan[1]*

1 Department of Biology, Lomonosov Moscow State University, 119234 Moscow, Russia
2 Laboratory of Structural-Functional Organization of Chromosomes, Institute of Gene Biology, Russian Academy of Sciences, 119334 Moscow, Russia
3 Faculty of Biology, MSU-BIT Shenzhen University, Shenzhen, China
4 Department of Physics, Lomonosov Moscow State University, 119234 Moscow, Russia
5 Shemyakin-Ovchinnikov Institute of Bioorganic Chemistry, Russian Academy of Sciences, Moscow 117997, Russia
6 Fox Chase Cancer Center, Philadelphia, PA 19111, USA
† - These authors contributed equally
* Correspondence: shaytan_ak@mail.bio.msu.ru ; Tel.: +7-(495)-939-5738

Supplementary Figures

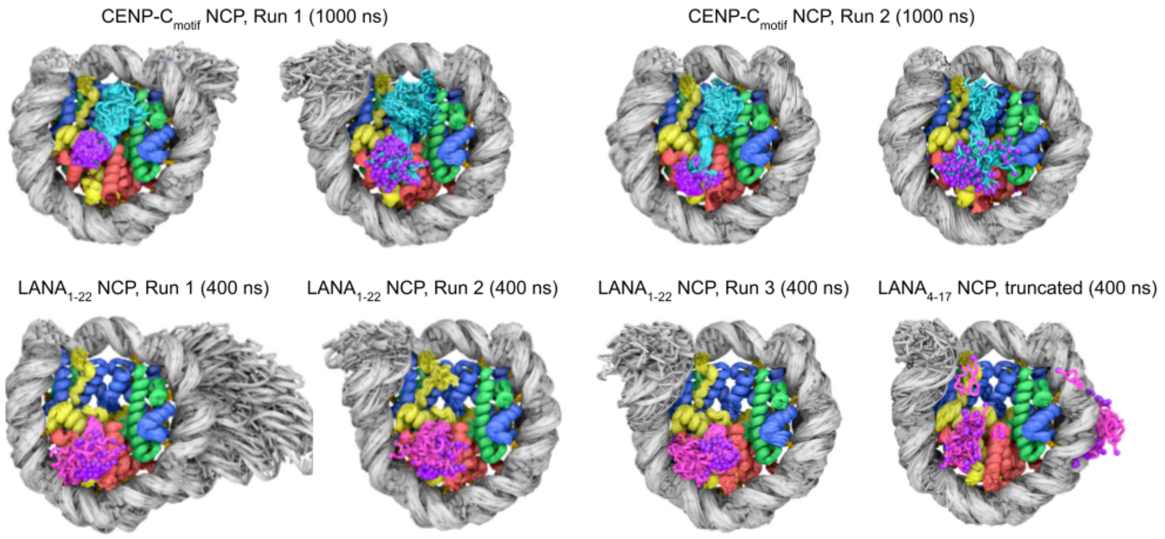

Supplementary Figure S1.1. Overview of MD simulation of $LANA_{1-22}$ and $CENP\text{-}C_{motif}$ with NCP. Overlay of MD snapshots spaced 10 ns apart.

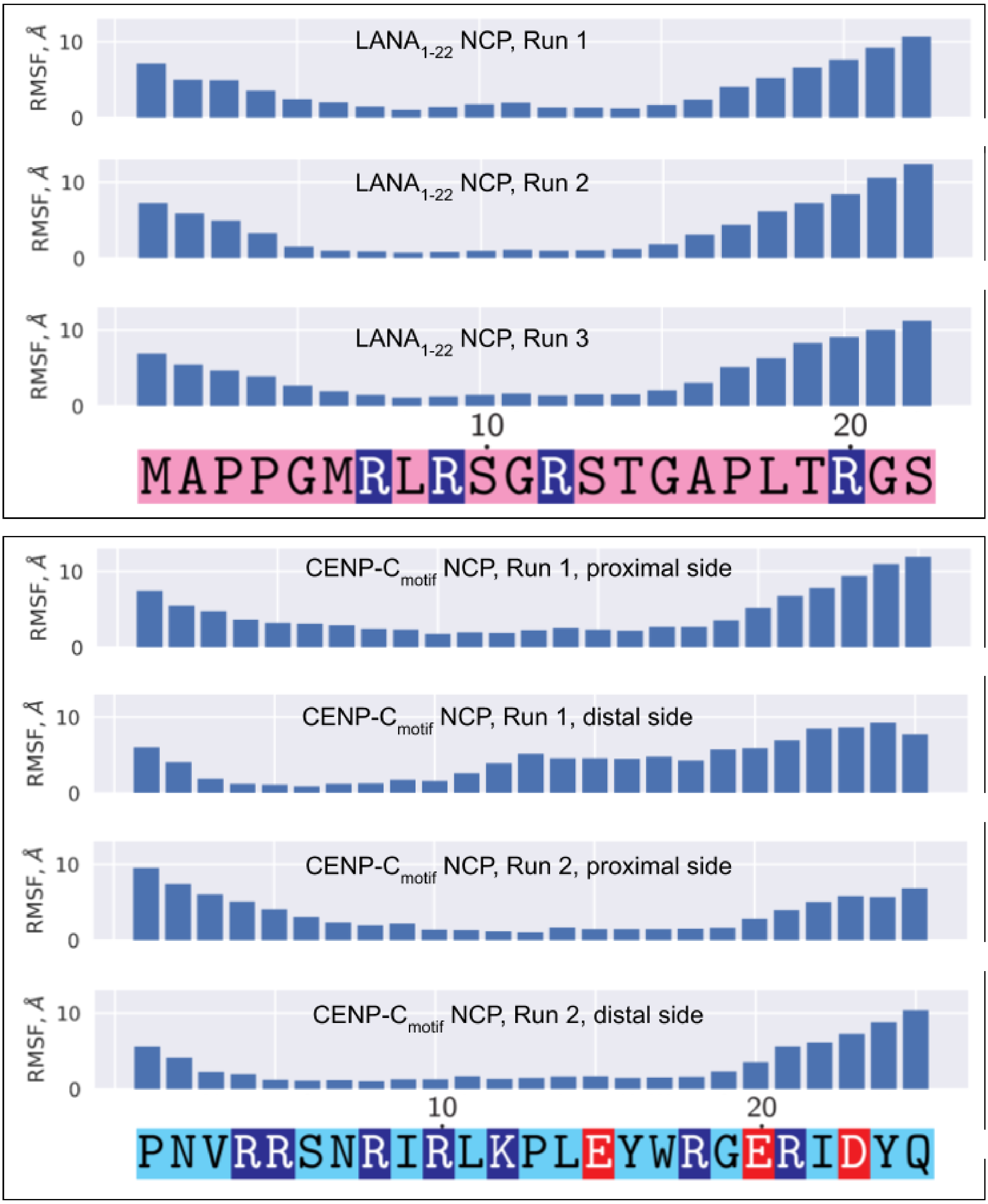

Supplementary Figure S1.2. Root-mean-square fluctuations of peptides' Cα-atom positions In MD simulation of the NCP with LANA$_{1\text{-}22}$ and CENP-C$_{motif}$. Trajectories of peptides were aligned by superimposing the H2A-H2B dimer histone folds (Cα-atom positions of α1, α2 and α3-helices).

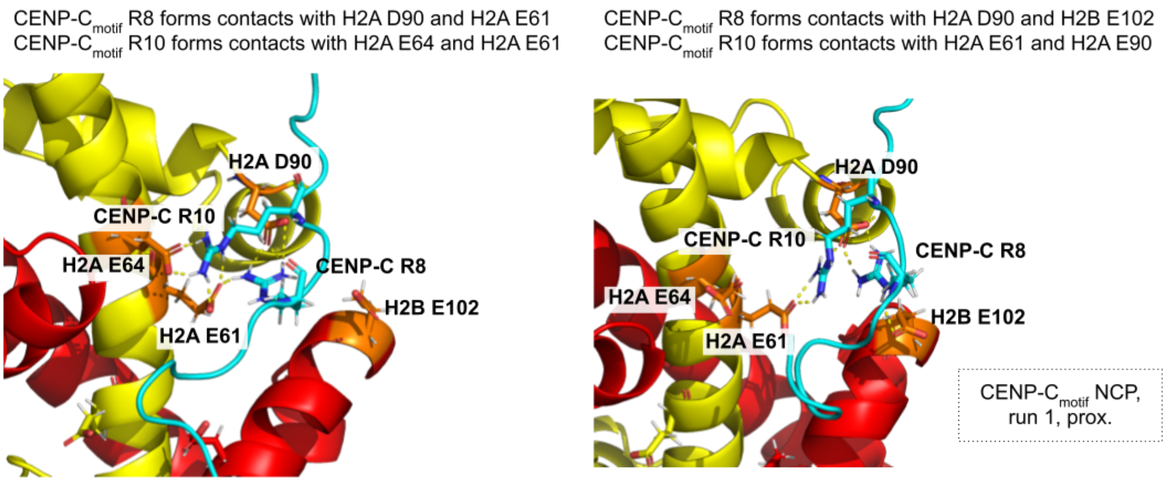

Supplementary Figure S1.3. Reorganization of CENP-C$_{motif}$ and NCP acidic patch contacts in MD simulation (run 1, proximal side). NCP acidic patch residues are colored in orange.

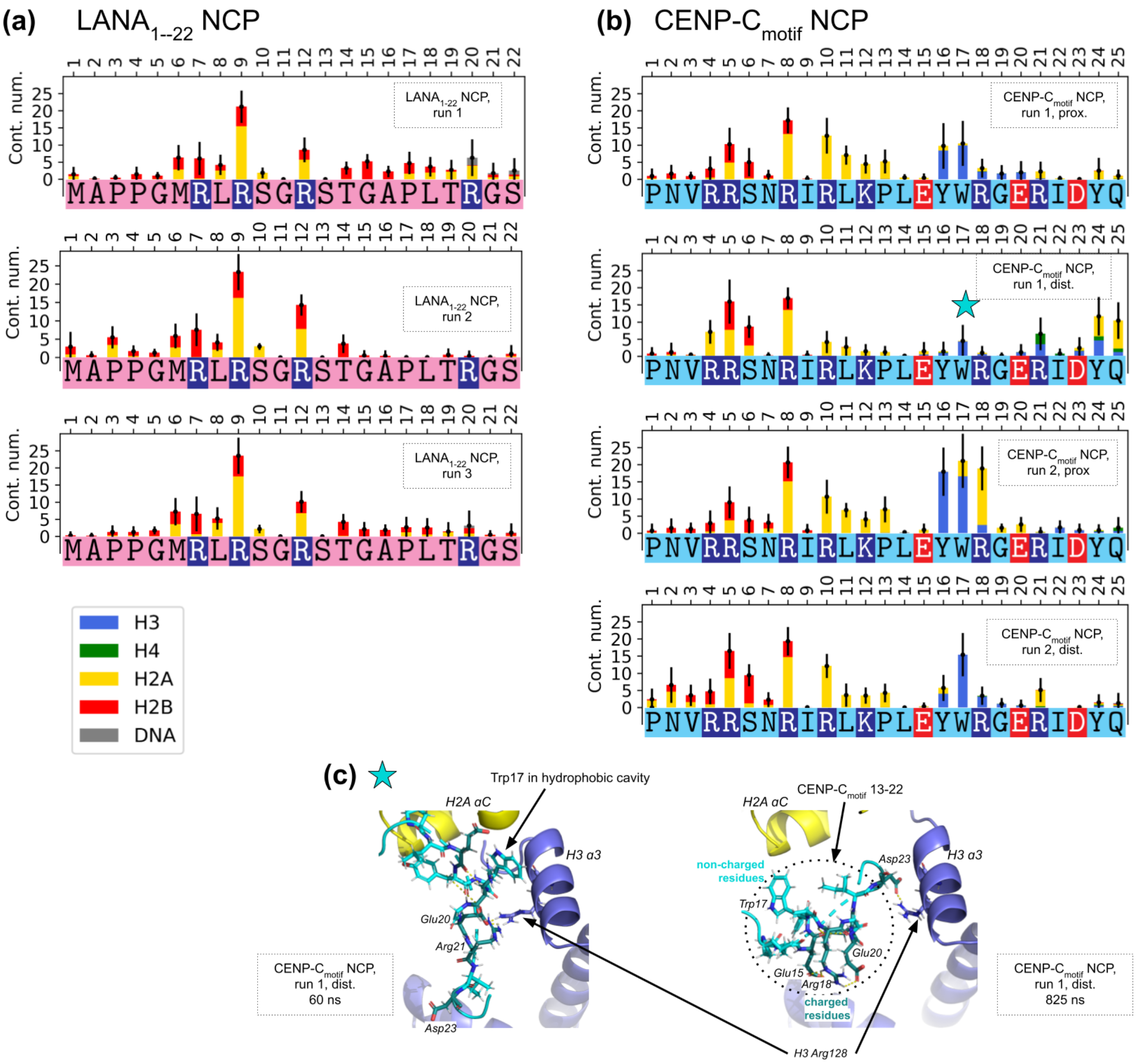

Supplementary Figure S1.4. Contacts profiles of $LANA_{1\text{-}22}$ (a) and CENP-C_{motif} (b) with the NCP. The trajectory average (for each trajectory) number of atom-atom contacts between peptide and NCP is shown on top of the sequence. (c) Reorganization of CENP-C which leads to the loss of hydrophobic contact between CENP-C_{motif} and H3 α3-helix.

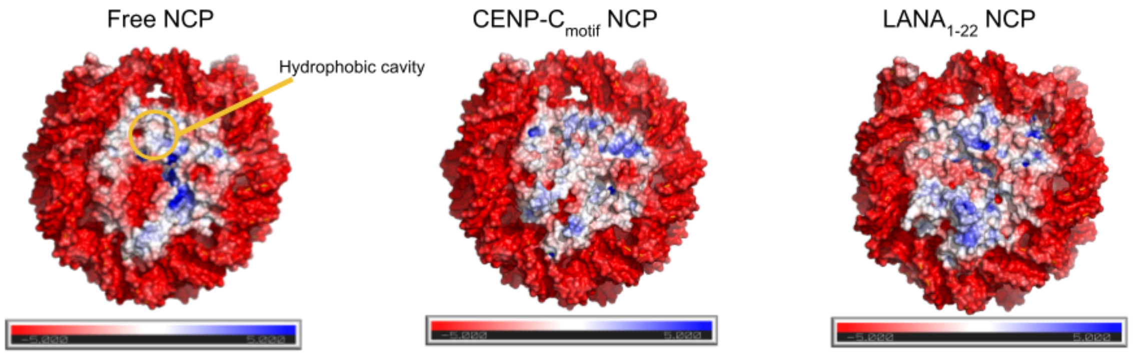

Supplementary Figure S1.5. Effects of CENP-C_{motif} and $LANA_{1\text{-}22}$ binding on NCP surface electrostatic potential. Electrostatic surface was calculated and visualized using Pymol and its APBS electrostatic calculation plugin.

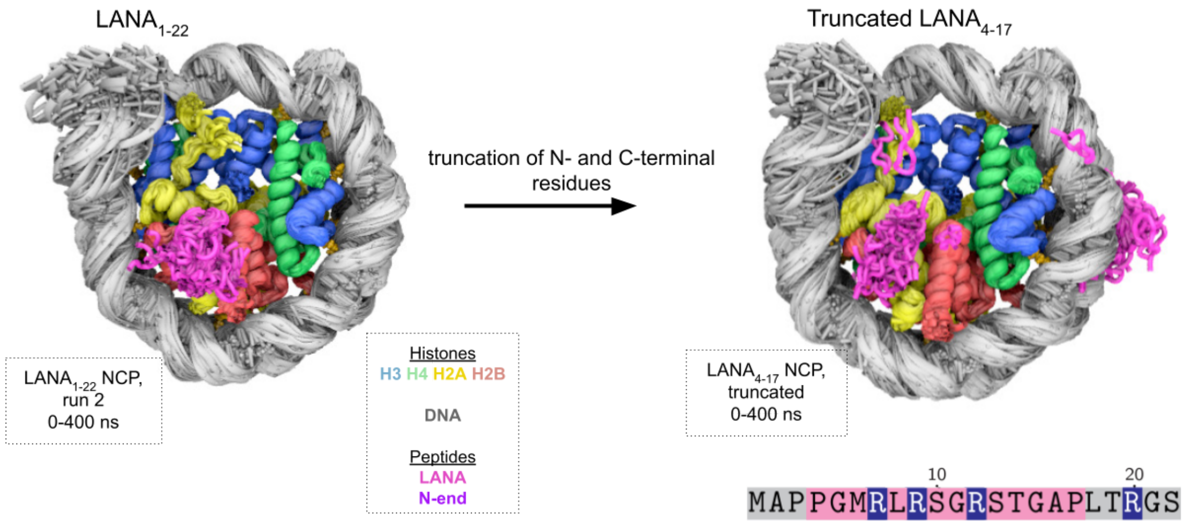

Supplementary Figure S1.6. Effects observed due to truncation of flexible ends of $LANA_{1\text{-}22}$ peptide on its dynamics and interactions with the NCP. Results of MD simulations are presented as an overlay of MD snapshots. Truncation regions correspond to unresolved residues in experimental structure (PDB ID 1ZLA) and are shaded gray on the $LANA_{1\text{-}22}$ sequence.

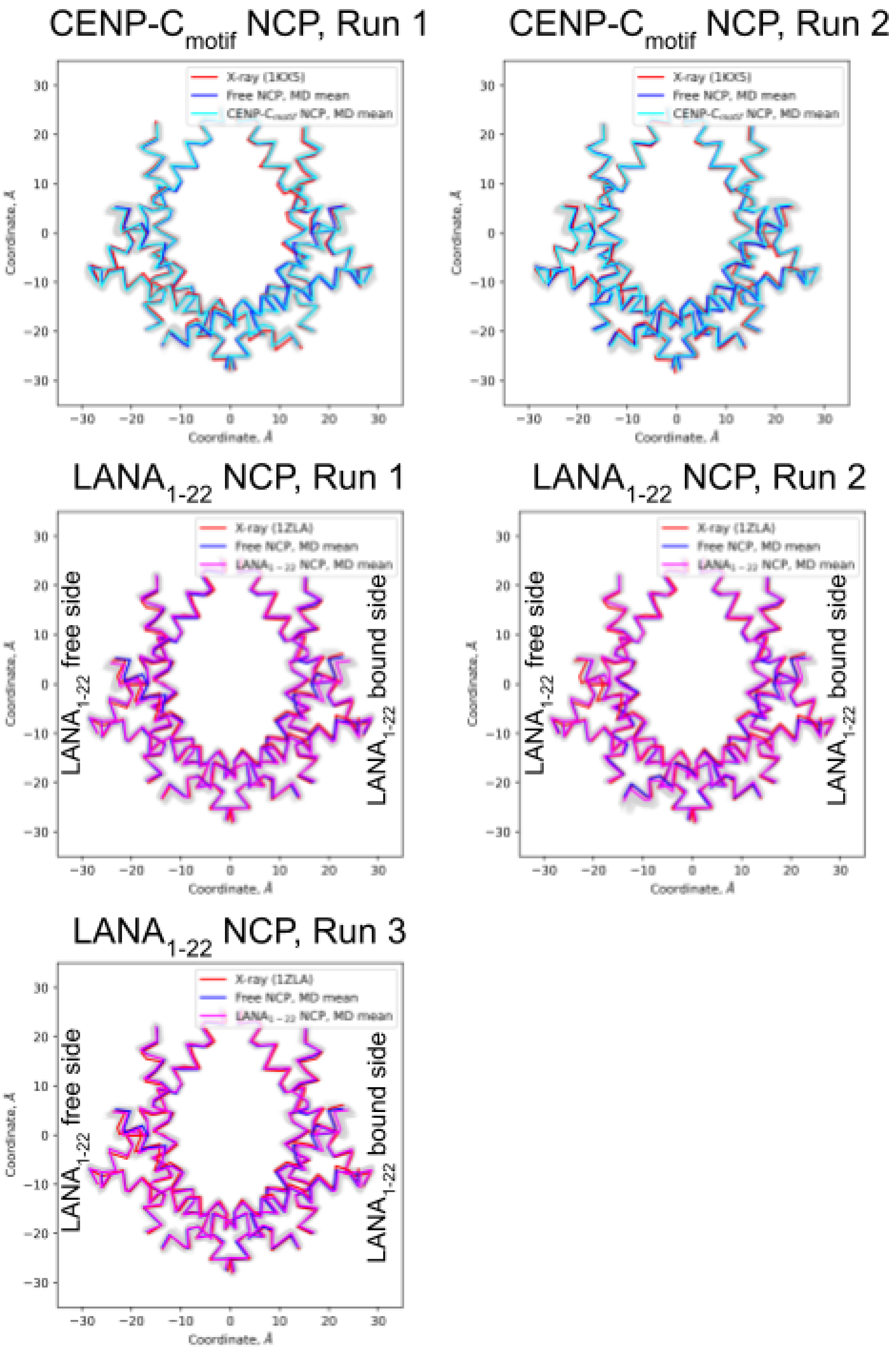

Supplementary Figure S2.1. 2D projections of histone α2-helices conformations (Cα-atom positions) in MD simulations of NCPs bound to peptides. The mean helix conformations and conformations in NCP X-ray structure are shown in color according to the legend. Gray lines represent MD Cα-atom positions in the corresponding trajectory.

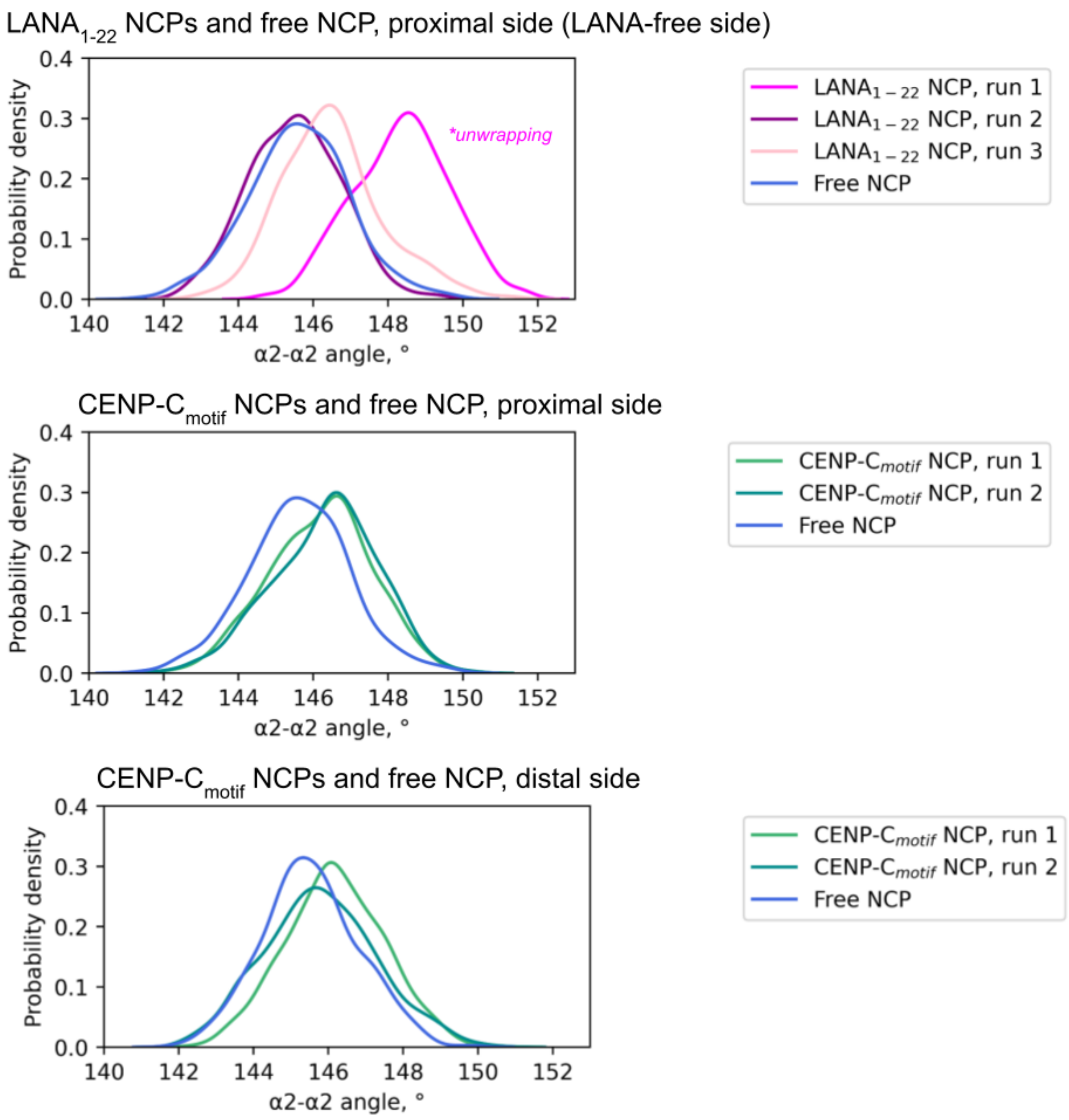

Supplementary Figure S2.2. Probability distributions of α2-α2 angle values during MD simulations of NCPs bound to peptides in comparison with MD of the free NCP$^{tt}_{147}$. Statistics are given in Supplementary Table 4.1, median values are in Supplementary Table 4.3.

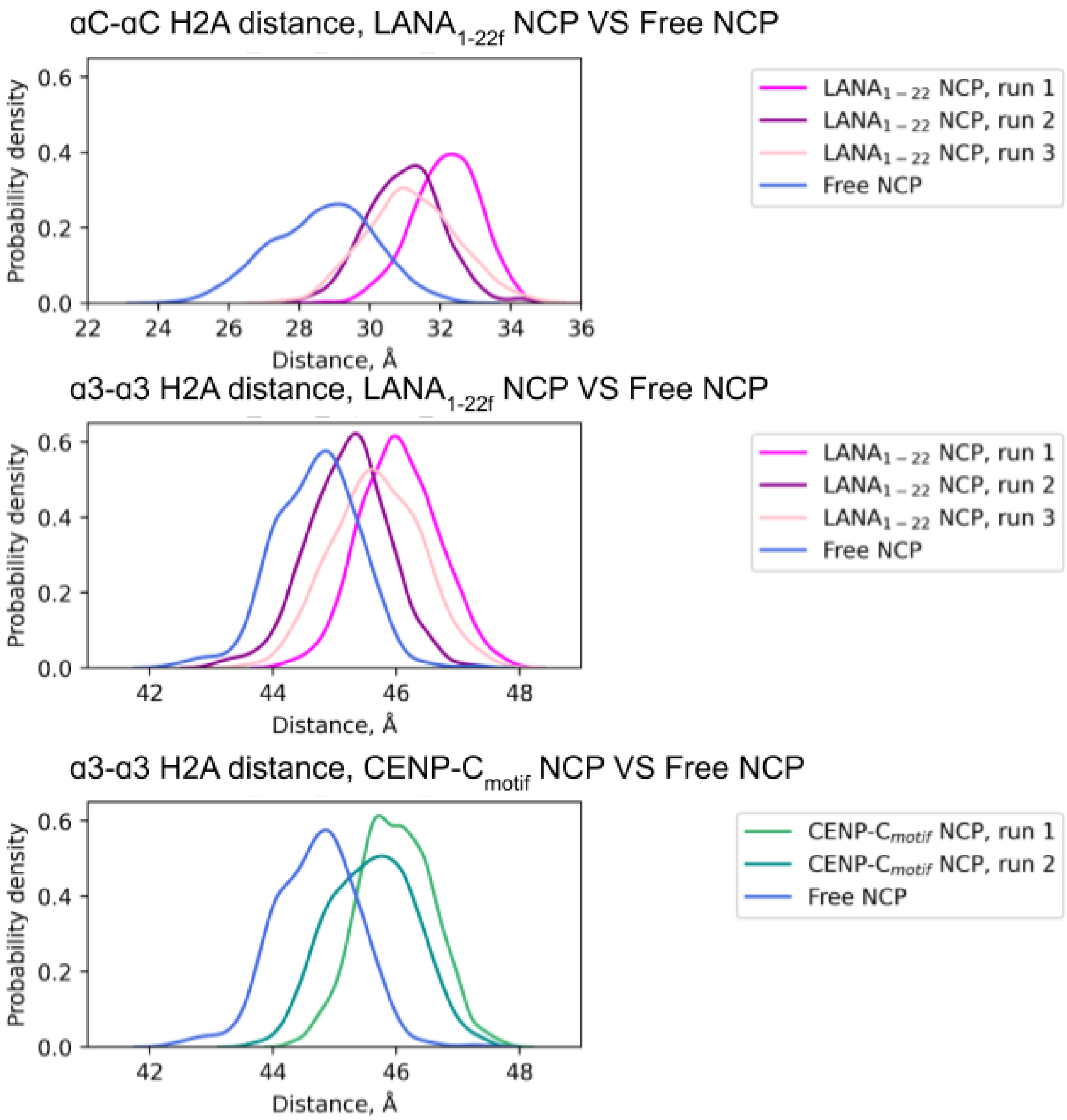

Supplementary Figure S2.3. Probability distributions of distance between α3-α3 H2A helices and αC-αC H2A helices. Values from MD simulations of NCP bound to peptides in comparison with MD of the free NCP^{tt}_{147} are shown. Statistics are given in Supplementary Table 4.2, median values are in Supplementary Table 4.3.

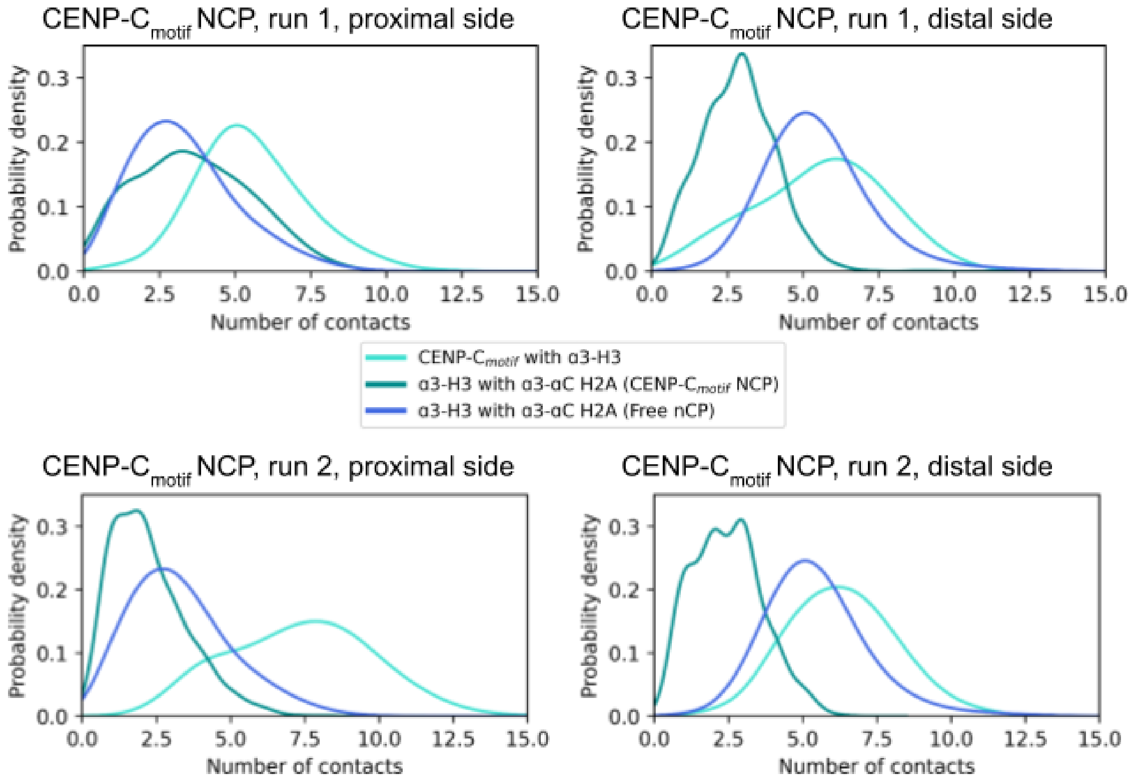

Supplementary Figure S2.4. Comparison of number of contacts between α3-αC H2A, α3 H3 and CENP-C$_{motif}$ in MD simulation of free NCP and NCP bound to CENP-C$_{motif}$.

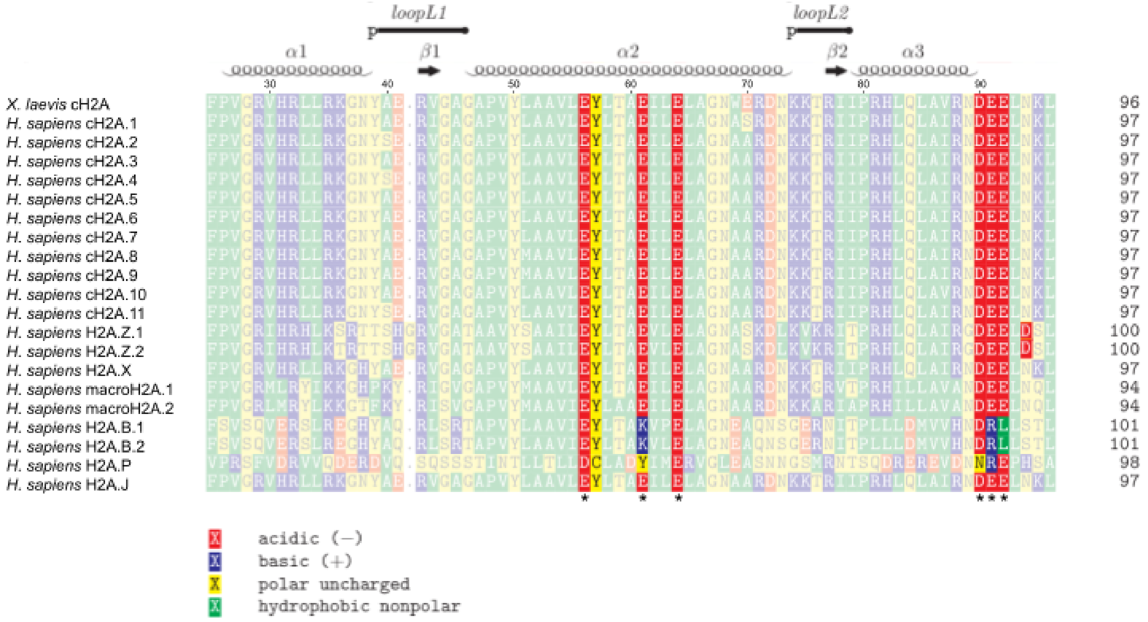

Supplementary Figure S3.1. Multiple sequence alignment of human sequences of H2A histone variants. Highlighted amino acids are the important contact sites on the histone octamer surface engaged during peptide binding. The color of the amino acids correspond to their hydropathicity: red - acidic, blue - basic, yellow - polar uncharged, green - hydrophobic nonpolar. Acidic patch amino acids are indicated by asterisks. HGNC gene names of aligned sequences are H2AC1, H2AC4, H2AC6, H2AC7, H2AC11, H2AC12, H2AC14, H2AC18, H2AC20, H2AC21, H2AC25, H2AZ1, H2AZ2, H2AX, MACROH2A1, MACROH2A2, H2AB1, H2AB2, H2AP and H2AJ.

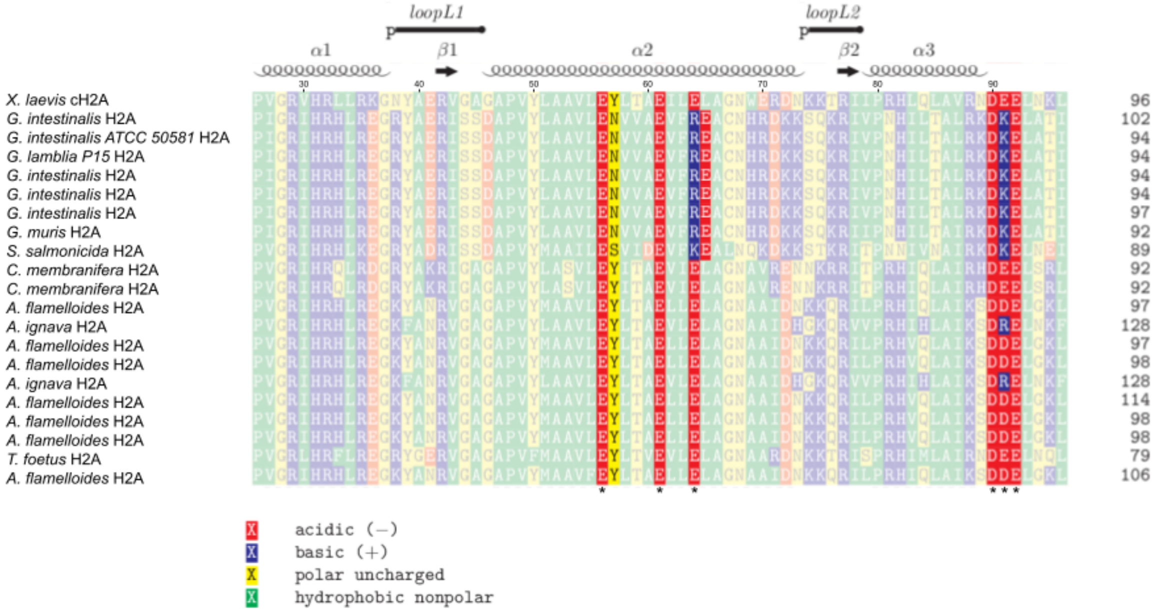

Supplementary Figure S3.2. Multiple sequence alignment of H2A histones from species belonging to Metamonada phylum. Highlighted amino acids are the important contact sites on the histone octamer surface engaged during peptide binding. The color of the amino acids correspond to their

hydropathicity: red - acidic, blue - basic, yellow - polar uncharged, green - hydrophobic nonpolar. Acidic patch amino acids indicated by asterisks.

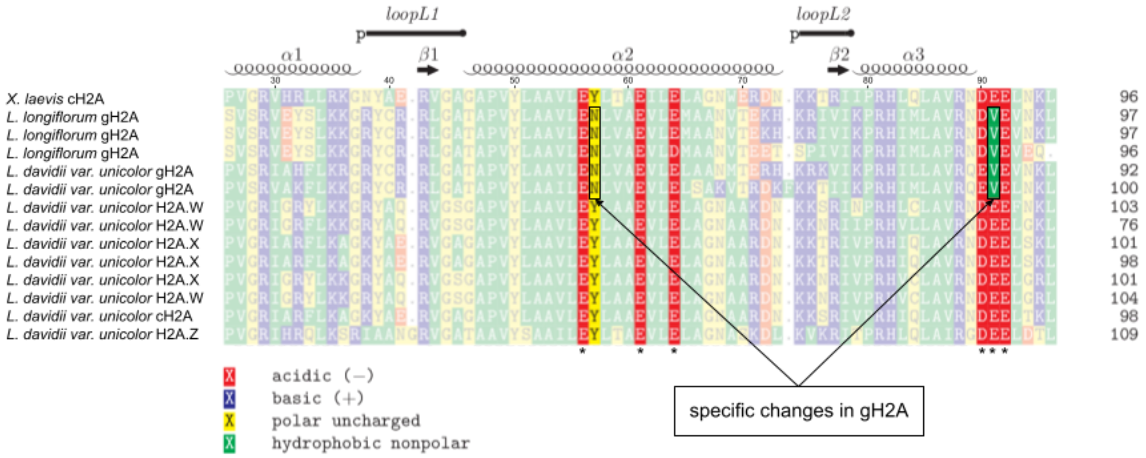

Supplementary Figure S3.3. Multiple sequence alignment of H2A histone variant sequences from genus *Lilium*. Highlighted amino acids are the important contact sites on the histone octamer surface engaged during peptide binding. The color of the amino acids correspond to their hydropathicity: red - acidic, blue - basic, yellow - polar uncharged, green - hydrophobic nonpolar. Acidic patch amino acids indicated by asterisks.

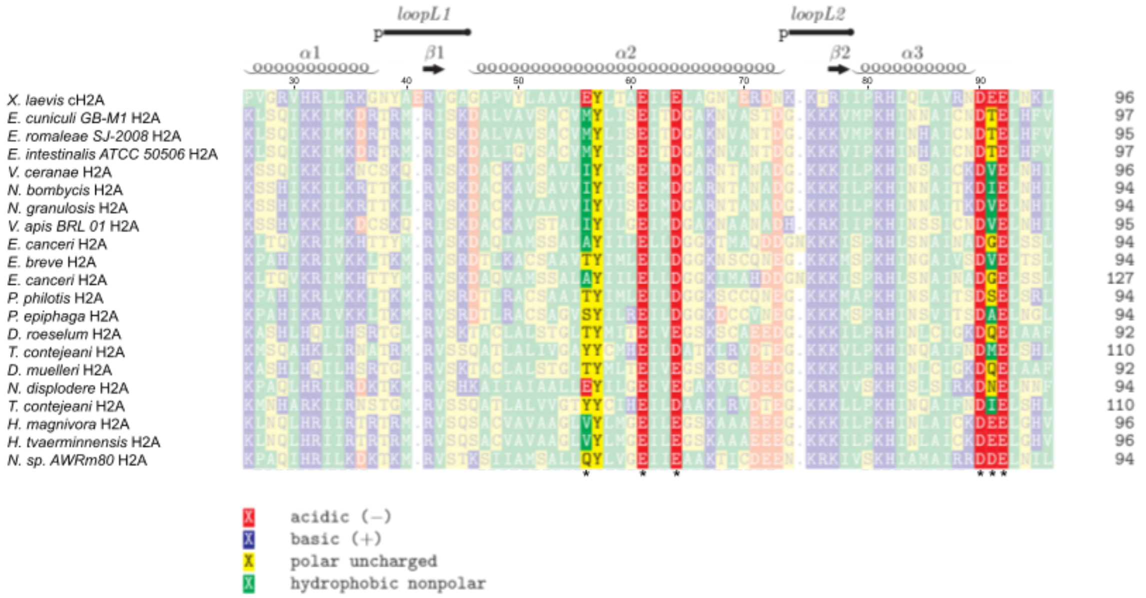

Supplementary Figure S3.4. Multiple sequence alignment of H2A histone variant sequences from microsporidia. Highlighted amino acids are the important contact sites on the histone octamer surface engaged during peptide binding. The color of the amino acids correspond to their hydropathicity: red - acidic, blue - basic, yellow - polar uncharged, green - hydrophobic nonpolar. Acidic patch amino acids indicated by asterisks.

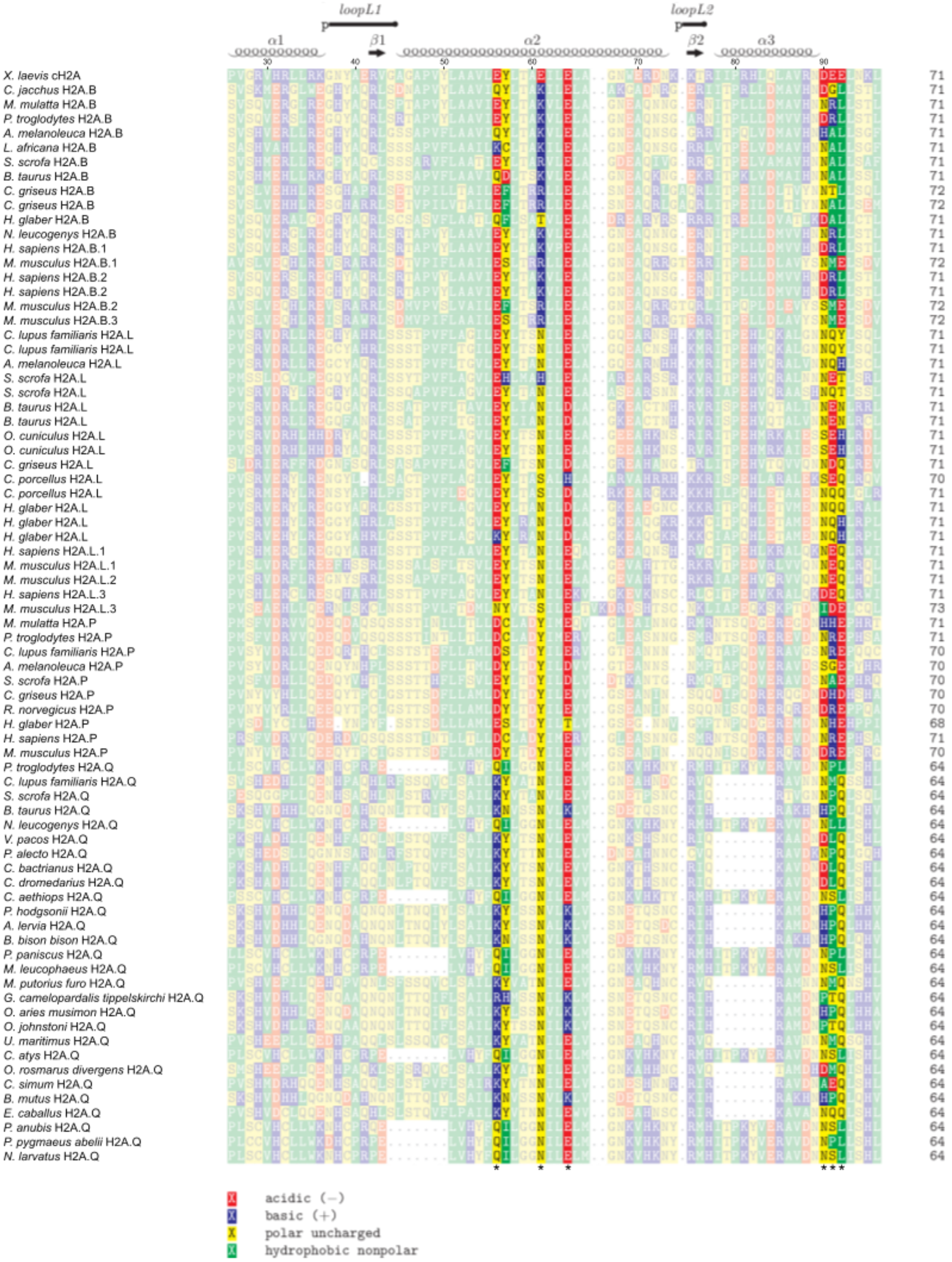

Supplementary Figure S3.5. Multiple sequence alignment of mammalian short H2A histone sequences. Highlighted amino acids are the important contact sites on the histone octamer surface engaged during peptide binding. The color of the amino acids correspond to their hydropathicity: red - acidic, blue - basic, yellow - polar uncharged, green - hydrophobic nonpolar. Acidic patch amino acids indicated by asterisks.

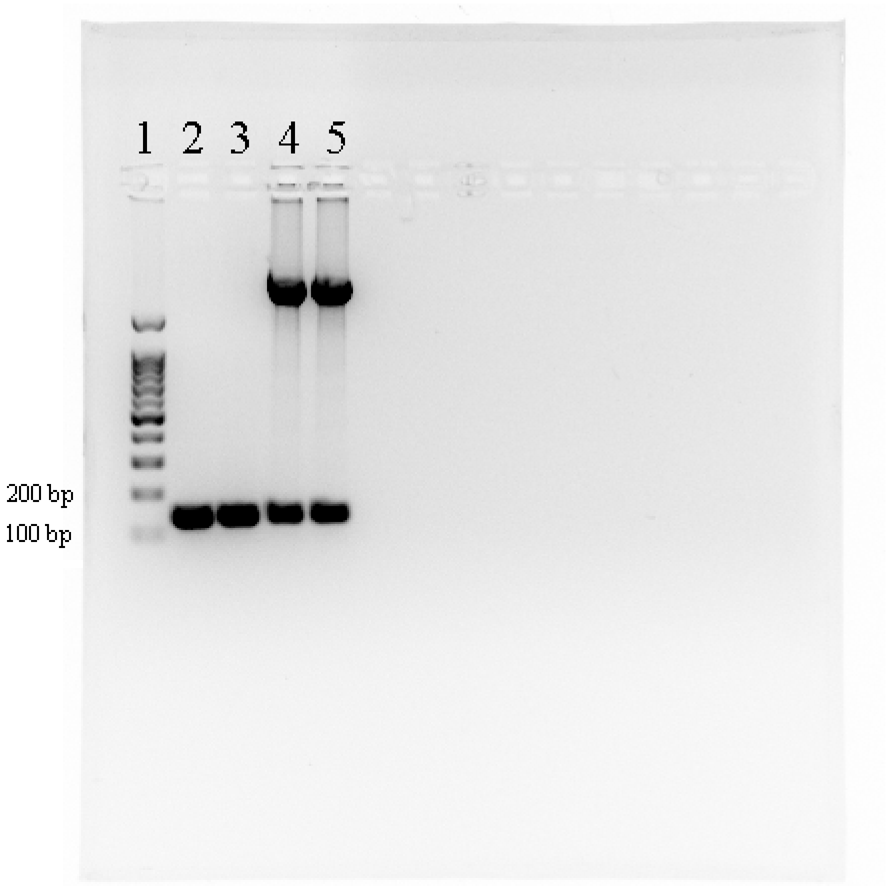

Supplementary Figure S4.1. A PAGE analysis of the DNA fragments prepared for the assembly of nucleosomes. Lane 1 - 100 bp DNA ladder marker, 2-3 - Purified nucleosomal DNA (cleaned from the vector backbone), 4-5 - Nucleosomal DNA and the vector backbone.

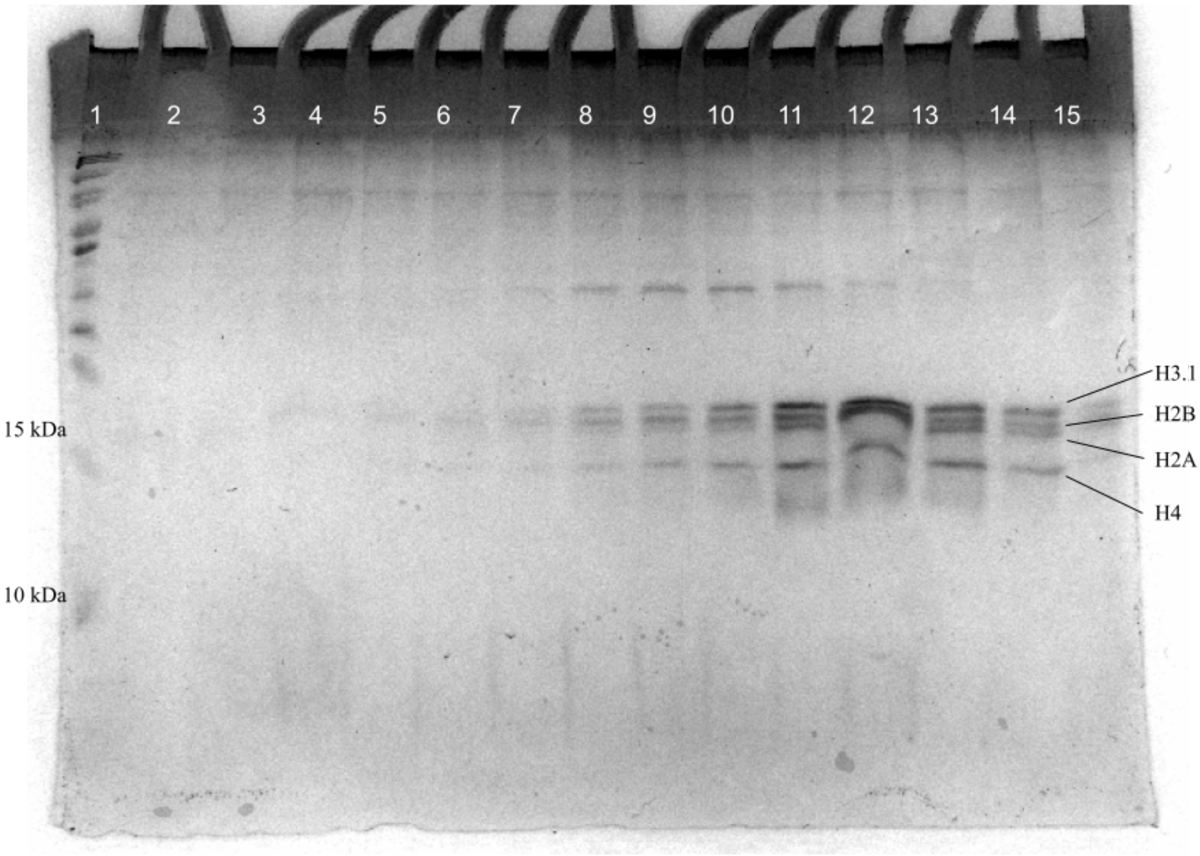

Supplementary Figure S4.2. A PAGE analysis of gel filtration fractions containing histone octamers.

Lane 1 - Protein marker, 2-15 - Fractions after gel filtration, 11-15 - Fractions containing octamers taken for nucleosome assembly.

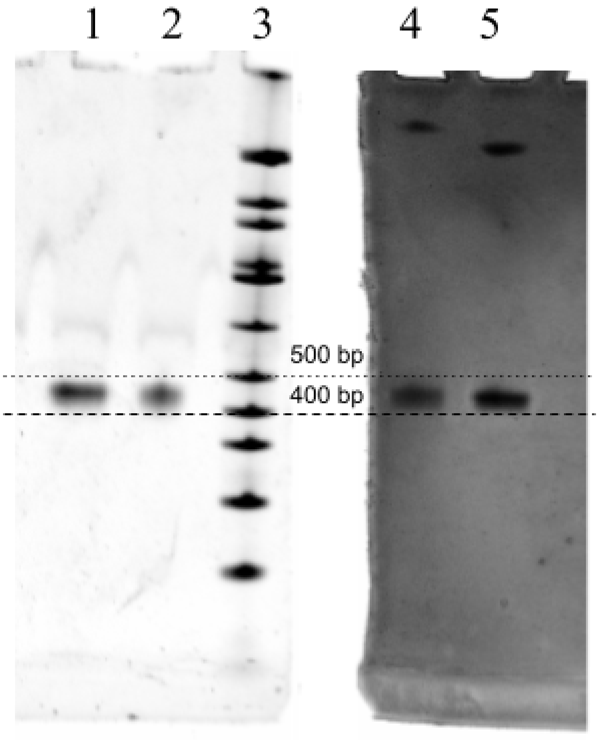

Supplementary Figure S4.3. Nucleosome quality assessment using 6% PAGE.
Lanes 1-2 - Nucleosomes (stained with SYBR Gold), 3 - 1000 bp DNA ladder marker (stained with SYBR Gold), 4-5 - Nucleosomes (stained with Coomassie Brilliant Blue).

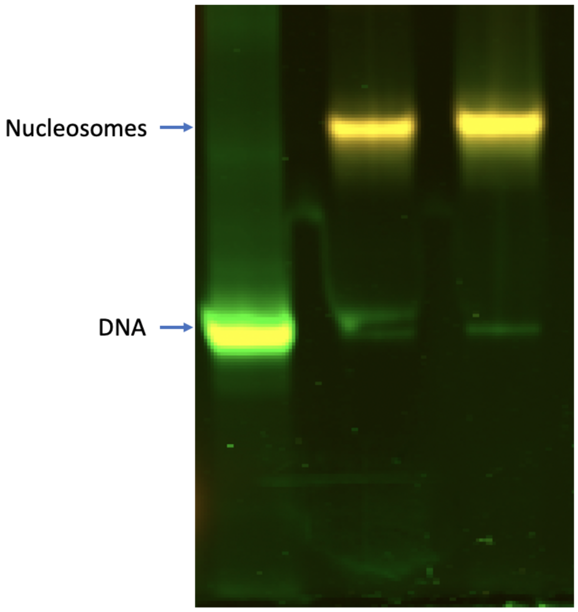

Supplementary Figure S5. PAGE analysis of assembled fluorescently labeled nucleosomes for spFRET. Yellow bands - nucleosomes, green bands - free DNA. Two scans using Typhoon scanner (GE Healthcare, USA) in Cy3 and Cy5 channels were merged.

Supplementary Tables

Supplementary Table S1. Systems simulated in MD simulations.
List of systems used in MD simulations and achieved trajectory times. PDB ID is the identifier of the structure that was used as the initial structure for MD simulations. The initial structures were altered by truncating histone tails and reconstructing missing histone residues, DNA and peptide residues where necessary.

System name	PDB ID	Peptide copies per NCP, chain ID	Atom number	Water molecules	Ion number, Na/Cl	Minimum periodic image distance, nm	Time, ns
$LANA_{1\text{-}22}$ NCP, 1 run	1ZLA	1 (K)	213K	64K	393/173	1.04	400
$LANA_{1\text{-}22}$ NCP, 2 run	1ZLA	1 (K)	213K	64K	393/173	1.48	400
$LANA_{1\text{-}22}$ NCP, 3 run	1ZLA	1 (K)	213K	64K	393/173	1.01	400
$LANA_{4\text{-}17}$ NCP, truncated	1ZLA	1 (K)	167K	48K	353/132	0.17*	400
$CENP\text{-}C_{motif}$ NCP, run 1	4X23/ 1KX5	2 (W,X)	214K	64K	394/174	1.20	1000
$CENP\text{-}C_{motif}$ NCP, run 2	4X23/ 1KX5	2 (W,X)	214K	64K	394/174	2.74	1000

* LANA peptide dissociated from one NCP and reassociated with its periodic image

Supplementary Table S2a. Statistical significance analysis of the difference between H2A-H2B dimer α2-α2-angle values in MD simulations of free NCP and $LANA_{1\text{-}22}$ bound NCP. Distal (LANA-binding) dimer was analyzed.

Systems used for comparison	two-sample Kolmogorov-Smirnov test		T-test for the means of two independent samples	
	statistic	p-value	statistic	p-value

Free NCP - LANA$_{1-22}$ NCP (run 1)	0.63	<0.001	-10.57	<0.001
Free NCP - LANA$_{1-22}$ NCP (run 2)	0.69	<0.001	-12.38	<0.001
Free NCP - LANA$_{1-22}$ NCP (run 3)	0.51	<0.001	-8.55	<0.001
LANA$_{1-22}$ NCP (run 1) - LANA$_{1-22}$ NCP (run 2)	0.20	0.085	-2.39	0.018
LANA$_{1-22}$ NCP (run 1) - LANA$_{1-22}$ NCP (run 3)	0.19	0.124	1.56	0.121
LANA$_{1-22}$ NCP (run 3) - LANA$_{1-22}$ NCP (run 2)	0.28	0.003	3.79	<0.001
Free NCP - CENP-C$_{motif}$ NCP run 1	0.13	0.90	0.10	0.92
Free NCP - CENP-C$_{motif}$ NCP run 2	0.19	0.47	-0.92	0.36
CENP-Cmotif NCP run 1 - CENP-Cmotif NCP run 2	0.25	0.42	0.76	0.45

Supplementary Table S2b. Statistical significance analysis of the difference between H2A αC-αC-distance values in MD simulations of free NCP and CENP_C$_{motif}$ NCP.

Systems used for comparison	two-sample Kolmogorov-Smirnov test		T-test for the means of two independent samples	
	statistic	p-value	statistic	p-value
Free NCP - LANA$_{1-22}$ NCP, run 1	0.88	<0.001	-7.51	<0.001
Free NCP - LANA$_{1-22}$ NCP, run 2	0.66	<0.001	-4.61	<0.001
Free NCP - LANA$_{1-22}$ NCP, run 3	0.54	0.004	-3.89	<0.001
LANA$_{1-22}$ NCP, run 1 - LANA$_{1-22}$ NCP, run 2	0.52	0.006	-3.98	<0.001

LANA$_{1-22}$ NCP, run 2 - LANA$_{1-22}$ NCP, run 3	0.24	0.60	-0.14	0.89
LANA$_{1-22}$ NCP, run 1 - LANA$_{1-22}$ NCP, run 3	0.48	0.02	-3.10	0.004
Free NCP - CENP-C$_{motif}$ NCP, run 1	0.90	<0.001	-7.85	<0.001
Free NCP - CENP-C$_{motif}$ NCP, run 2	0.88	<0.001	-7.84	<0.001
CENP-C$_{motif}$ NCP, run 1 - CENP-C$_{motif}$ NCP, run 2	0.14	0.94	-0.10	0.92

Supplementary Table S2c. Median values of αC-αC H2A distance, α3-α3 H2A distance and α2-α2 H2A-H2B angles probability distributions.

System name	LANA$_{1-22}$ NCP			CENP-C$_{motif}$ NCP		Free NCP
Feature	run 1	run 2	run 3	run 1	run 2	
αC-αC H2A distance, Å	32.3	31.3	31.0	32.3	32.0	29.1
α3-α3 H2A distance, Å	46.0	45.4	45.6	45.7	45.8	44.9
α2-α2 H2A-H2B angle, proximal, °	148.5 (LANA-free)	145.6 (LANA-free)	146.4 (LANA-free)	146.6	146.6	145.5
α2-α2 H2A-H2B angle, distal, °	147.6	148.0	147.3	146.1	145.7	145.3

Supplementary Table S3. Sequences of synthesized peptides used in experimental studies. Note: for fluorescence polarization assay LANA$_{1-22}$ peptide had a fluorescent FAM label attached at its N-terminus.

	Number of amino acids	Amino acid sequence	Molecular weight, kDa	Charge (pH=8)
LANA$_{1-22}$	22	MAPPGMRLRSGRSTGAPLTRGS	2,256	3,942

CENP-C_{motif}	25	PNVRRSNRIRLKPLEYWRGERIDY Q	3,215	3,979

Supplementary Materials and Methods

Nucleosome preparation

The preparative isolation of the plasmids containing eight repeats of the high-affinity 147 bp Widom 603 sequence was carried as described in [1]. The purified plasmids were incubated for 16 hours at 37 °C with EcoR V restriction enzyme (300 units per 1 mg of plasmid DNA), then they were incubated for another 2 hours at 37 °C in shaker (220 rpm) for more effective restriction.

The Widom 603 fragments were separated from the parent plasmid by polyethylene glycol precipitation in order to remove the vector backbone. 0.374 volume of 40% PEG 6000 and 0.200 volume of 4 M NaCl was added to the restriction mixture (this resulting concentration of PEG was 9.5% and NaCl was 0.5M).

After centrifugation (27,000 g for 20 minutes at 4 °C), the Widom 603 fragment was present only in the aqueous phase and the vector backbone was present in the sediment. DNA from the aqueous phase was precipitated by adding 0.1 volume of 4 M NaCl and 2.5 volume of cold 100% ethanol. After which centrifugation was carried out (at 27,000 g for 30 minutes at 4 °C). After this, the precipitated DNA was transferred to TE 10/0.1 buffer (10mM Tris-Cl pH 8.0, 0.1 mM EDTA) and stored at -20 °C.

Evaluation of the quality of purification of the target fragment from the non-restricted vector backbone was done using PAGE in 2% agarose gel in a TAE buffer (see Supplementary Figure S6.1).

After purification of single histones using ion exchange chromatography (using HiTrap Q FF anion exchange chromatography column (Cytiva) and HiTrap SP Sepharose FF (Cytiva) cation exchange chromatography column) and histone octamer assembly (as described in [1]), purification of histone octamers was performed using gel filtration. A HiLoad 16/600 Superdex 200 pg column (Cytiva) was used for gel filtration. To assemble nucleosomes, octamer fractions containing the least amount of protein impurities were used. Denaturing SDS-PAGE with 18% gels was performed to analyze gel filtration fractions (Supplementary Figure S6.2).

PAGE with 4% gels was used to check the quality of nucleosomes (electrophoresis buffer: 10 mM HEPES-Na pH 8.0, 0.2 мM EDTA, 10% glycerol). Nucleosomes are composed of DNA and histones. Therefore, the gel was stained with SYBR Gold (for DNA staining) and Coomassie Brilliant Blue (for protein staining) (Supplementary Figure S6.3).

References

1. Dyer, P.N.; Edayathumangalam, R.S.; White, C.L.; Bao, Y.; Chakravarthy, S.; Muthurajan, U.M.; Luger, K. Reconstitution of Nucleosome Core Particles from Recombinant Histones and DNA. In *Methods in Enzymology*; Elsevier, 2003; Vol. 375, pp. 23–44 ISBN 978-0-12-182779-3.
